# Role of Mitochondria Transfer in Infertility: A Commentary

**DOI:** 10.3390/cells11121867

**Published:** 2022-06-08

**Authors:** Cristina Rodríguez-Varela, Elena Labarta

**Affiliations:** 1 IVI Foundation, Instituto de Investigación Sanitaria La Fe (IIS La Fe), 46026 Valencia, Spain; elena.labarta@ivirma.com; 2 IVI RMA Valencia, 46015 Valencia, Spain

**Keywords:** mitochondria, infertility, poor oocyte quality, poor embryo quality, in vitro fertilization, mitochondria transfer, advanced maternal age, autologous, heterologous

## Abstract

Mitochondria transfer techniques were first designed to prevent the transmission of diseases due to mutations in mtDNA, as these organelles are exclusively transmitted to the offspring by the oocyte. Despite this, given the crucial role of mitochondria in oocyte maturation, fertilization and subsequent embryo development, these approaches have been proposed as new potential strategies to overcome poor oocyte quality in infertile patients. This condition is a very common cause of infertility in patients of advanced maternal age, and patients with previous in vitro fertilization (IVF) attempt failures of oocyte origin. In this context, the enrichment or the replacement of the whole set of the oocyte mitochondria may improve its quality and increase these patients’ chances of success after an IVF treatment. In this short review, we will provide a brief overview of the main human studies using heterologous and autologous mitochondria transfer techniques in the reproductive field, focusing on the etiology of the treated patients and the final outcome. Although there is no current clearly superior mitochondria transfer technique, efforts must be made in order to optimize them and bring them into regular clinical practice, giving these patients a chance to achieve a pregnancy with their own oocytes.

## 1. Introduction

Mitochondria transfer involves a range of several techniques in which mitochondria from a donor (heterologous transfer) or from the patient (autologous transfer) are transferred into the patient’s cells. In the reproductive field, the main target cell is the female gamete: the oocyte [1]. 

In this field, mitochondria transfer was first mainly designed to prevent the transmission of diseases due to mutations in mtDNA [2,3,4]. Mitochondria are exclusively transmitted to the offspring by the oocyte, as practically the sperm’s only contribution is genetic material [5]. Hence, any detrimental mutation in the oocyte mtDNA will lead to a mitochondrial-related disease in the offspring. In this context, mitochondria transfer has been proposed as a feasible alternative to avoid the transmission of the patient’s damaged organelles.

Nevertheless, mitochondria are also important organelles involved in the acquisition of optimal oocyte quality, proper fertilization and subsequent embryo development. These properties arise from their role in energy production, Ca^2+^ homeostasis, oxidative stress management and apoptosis regulation, among others, essential for the proper execution of all the biological processes previously mentioned [6]. Hence, improving oocyte quality by means of enhancing mitochondrial quality has arisen as a novel strategy to improve the success of in vitro fertilization (IVF) treatments in women with a history of poor oocyte quality, women of advanced maternal age or patients with previous IVF failures, all of them sharing defects at the oocyte level.

In this context, these techniques can give rise to a replacement or merely an enrichment in mitochondrial content. The first would try to improve oocyte quality by replacing the whole set of the cell’s mitochondria, and the second will do so by increasing the number of healthy organelles within the oocyte. 

In addition, not only mitochondria can be transferred into the target oocyte. Some of these techniques involve the transfer of a cytoplasm portion in order to transfer mitochondria. This cytoplasm includes RNAs, proteins, energy-producing components and many other yet undetected factors that may also contribute to the enhancement of oocyte quality [7].

Mitochondria transfer techniques are conducted with this aim in the animal model, proving promising results in several studies [8,9,10,11,12]. These studies have laid the groundwork for the design and implementation of these techniques in human clinical studies, on which we will focus in this review. 

**Figure 1:**
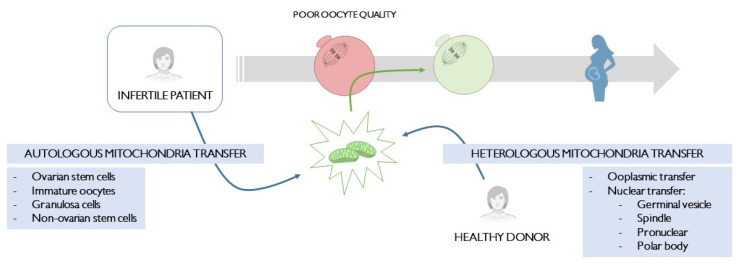
Schematic representation of mitochondria transfer techniques in order to improve poor oocyte quality in infertile patients. Autologous and heterologous techniques are specified.

In the following sections, a short review of different human studies performed in the reproductive field trying to enhance oocyte quality will be conducted. These studies will be divided according to mitochondrial source into two main categories (Figure 1). 

## 2. Heterologous Mitochondria Transfer

Heterologous mitochondria transfer aims to enhance the patient’s oocyte performance with mitochondria from a donor. 

To accomplish this, a fraction of the donor’s oocyte cytoplasm is transferred into the patient’s gamete in the ooplasmic transfer or cytotransfer, enriching it in healthy mitochondria [13]. On the other hand, the patient’s genetic material is transferred into an enucleated donor’s oocyte in the nuclear transfer, replacing the whole mitochondria content and trying to reduce to a minimum the amount of the patient’s own organelles left behind [1]. Nevertheless, in both scenarios, other cytoplasm components are transferred along with mitochondria during the procedure. 

However, the unknown deleterious effect of the interaction between the patient’s mtDNA, nuclear DNA and the donor’s mtDNA has led to some criticism regarding these techniques [14]. In recent years, research has focused on their optimization, trying to reduce as much as possible the amount of the patient’s mtDNA transferred in nuclear transfer techniques. Yet, ooplasmic transfer always involves the interaction of three different DNAs. A summary of the technical properties, advantages and disadvantages of all these techniques can be found in Rodríguez-Varela et al. 2021 [1]. The main human studies using these techniques will be further discussed in the following lines, and a summary of them is present in Table 1.

### 2.1. Ooplasmic Transfer (Cytotransfer)

Ooplasmic transfer or cytotransfer consists of the transfer of a small portion of the donor’s oocyte cytoplasm into the patient’s oocyte [13]. The first birth using this approach in humans was reported in 1997 [15]. Since then, this technique has been successfully conducted in humans [13,16,17,18,20]. Despite this, the proven heteroplasmy present not only in oocytes originated by this approach but also in embryonic material [16,31] and healthy live births [32] led to several ethical and technical concerns, and its use was even suspended in the United States in 2001 by the Food and Drug Administration (FDA) [33]. 

Despite the huge controversy regarding this technique, healthy offspring have been reported after cytotransfer in patients with a history of poor oocyte quality [20] and repeated implantation failure [17,18,19]. 

In 2021, cytotransfer was proved to improve oocyte quality of patients with low ovarian function, either due to advanced maternal age, low ovarian reserve or low ovarian response to stimulation, regardless of their age. Fertilization rates and early embryo development were enhanced after cytotransfer in this population. These effects may be due in part to its positive effects on the overall cytoplasmic function [20], which have also been shown to be improved in several studies [34]. Nevertheless, further human studies evaluating molecular parameters after ooplasmic transfer are important to better understand its effects at the molecular level and demonstrate its safety. 

Cytotransfer was also able to achieve pregnancy in patients with repeated implantation failure, regardless of whether this outcome was associated with poor embryo development [18,19] or not [17]. 

In addition to these promising results, an important advantage of this mitochondria enrichment technique is its feasibility. The donor’s cytoplasm can be injected into the patient’s oocyte along with the spermatozoa at the time of the ICSI procedure [20], making it part of the routine clinical practice in the IVF lab and accessible to all embryologists. 

### 2.2. Nuclear Transfer

Nuclear transfer consists of the transfer of the patient’s genetic material, in its different forms regarding the oocyte stage, into the donor’s cytoplasm previously enucleated [3,21,24,28]. If correctly performed, this technique can reduce to a minimum the amount of mtDNA carryover from the patient, leading to the interaction of only two different DNAs in the resulting oocyte, even though of different origin. 

However, one of its main limitations is its technically demanding protocol, which needs highly qualified and experienced embryologists performing the technique in order to achieve high success rates while reducing mtDNA carryover to a minimum. 

Nuclear transfer techniques can be divided into the cell stage in which the mitochondria replacement takes place. Human studies have been performed using germinal vesicle, spindle, pronuclear and polar body transfer, while blastomere transfer has only been conducted in the animal model [1]. 

#### 2.2.1. Germinal Vesicle Transfer

Germinal vesicle (GV) transfer is particularly interesting in women of advanced maternal age. The enhancement of mitochondrial function prior to completion of the first meiosis may prevent the high aneuploidy rates characteristic of this type of patient [35]. However, the main disadvantage of this approach is the required subsequent in vitro maturation of the GV to a metaphase II (MII) oocyte, a technique which still needs to be further optimized. 

The first GV transfer in humans was performed by Zhang’s group in 1999, transferring the nuclear material of oocytes from patients over 38 years old into enucleated young GVs. In this experiment, the overall oocyte reconstruction success rate was 20% (12/60). Despite the small sample size and the low efficiency of the technique, they showed how normal meiosis can occur after the transfer of a GV into an enucleated donor oocyte [21]. More recently, in 2020, Darbandi’s group tried a similar approach, although none of the oocytes fused [22]. 

#### 2.2.2. Spindle Transfer

Spindle transfer is less invasive than GV transfer, achieving lower mtDNA carryover rates [36]. Despite the suggested human oocyte sensitivity to this intervention [37], several studies have proven the feasibility of this technique in humans, as well as its low carryover [2,25]. The first live birth after spindle transfer was reported in 2017 by Zhang’s group [24]. 

This technique has been shown to significantly increase the number of good-quality blastocysts after the transfer of spindles from in vitro matured MII human oocytes, as a model of aged oocytes, into enucleated young fresh MII oocytes [23]. Thus, spindle transfer seems promising for the rescue of low-quality aged oocytes in patients of advanced maternal age. 

In line with improving embryo quality, Costa-Borges’ group is currently conducting the first registered pilot trial to overcome infertility with spindle transfer in 32–40 years old patients with a history of embryo developmental arrest [25]. Preliminary results of nine initial patients were presented at the ASRM annual meeting in 2020. They showed promising oocyte reconstruction success rates, as well as fertilization and good-quality blastocyst rates. These results indicate that spindle transfer-derived embryos are able to implant and sustain a healthy pregnancy to term in patients with a previous difficult reproductive history. 

#### 2.2.3. Pronuclear Transfer

The first pronuclear (PN) transfer in humans was performed in 2010, and it consisted of the relocation of the PN structures from abnormally fertilized oocytes into enucleated MII oocytes, proving successful onward development to the blastocyst stage and minimal mtDNA carryover [3]. However, the translation of this protocol into normally fertilized zygotes was not well tolerated [26]. 

In 2016, Hyslop’s group proved the feasibility of a novel PN transfer protocol that efficiently promoted the development to the blastocyst stage of reconstructed zygotes, based on the assumption that transplanting pronuclei shortly after completion of meiosis may be better than shortly before the first mitotic division [26]. Although Hyslop et al. conducted this experiment with zygotes derived from donated oocytes fertilized with donated sperm, in the same year, Zhang et al. reported a healthy pregnancy using this technique in a woman with a history of embryo developmental arrest [27]. 

Nevertheless, the main limitation in the applicability of this mitochondria transfer technique is the ethical concern of generating extra zygotes, which will be subsequently discarded [38]. 

#### 2.2.4. Polar Body Transfer

Polar body (PB) transfer might be the less invasive approach of all mitochondria transfer techniques, as PB are residual structures derived from oocyte meiotic divisions [39] and located outside the female gamete [4]. In addition, these structures are known to carry low mitochondrial content [40]. However, the proper residual nature of these structures and unknown consequences also constitute this technique’s main limitation.

Several studies have proven the correct de novo spindle formation after the first polar body transfer (PB1T) [28,30]. In 2017 the transfer of the first polar bodies, but not the second (PB2T), into enucleated in vitro matured donor metaphase II oocytes successfully generated normally fertilized zygotes with high efficiency for developing into blastocysts in a couple with a history of repeated embryo fragmentation, proven to be of maternal origin [29]. On the contrary, also in 2017, Ma’s group showed significantly lower blastocyst formation rates in the PB1T group in comparison to the control group in a population of healthy volunteers [28]. 

Despite the inefficient PB2T observed in Zhang et al., 2017 [29], in 2019, Tang’s group described a novel strategy for PB2T optimization, showing unaltered blastocyst quality in the PB2T and control groups [30]. This was tested in human in vitro matured oocytes. Thus, although promising, it requires further optimization in patients’ oocytes. 

In the case of PB2T, as well as with PN transfer, the generation of extra zygotes soon-to-be discarded constitutes the main ethical concern regarding this technique. 

## 3. Autologous Mitochondria Transfer

Autologous mitochondria transfer has arisen as an alternative to avoid the introduction of a third source of DNA in the oocyte. In general, these are mitochondria enrichment techniques, as their aim is to increase the number of healthy organelles within the oocyte, not to replace them. In addition, these techniques usually transfer solely mitochondria [41,42] instead of transferring other cytoplasm components into the oocyte. 

Human studies of autologous mitochondria transfer will be divided regarding the cell-type source of the mitochondria transferred. A summary of the main human studies using these techniques is presented in Table 2.

### 3.1. Ovarian Stem Cells

The presence of ovarian stem cells in the adult ovary was confirmed several years ago [46], although their contribution to postnatal oogenesis remains questionable [47]. Nevertheless, they constitute a source of high-quality germline autologous mitochondria from the same lineage of the oocyte.

In this context, a new protocol, so-called autologous germline mitochondrial energy transfer (AUGMENT^®^), was designed. This procedure involves the isolation of ovarian stem cells-derived mitochondria from the ovarian cortex obtained by laparoscopy, and their injection into the patient’s oocyte at the time of ICSI, along with spermatozoa [48]. 

However, ovarian stem cells constitute a difficult cell population to obtain and contain relatively few mitochondria [49]. In addition, they did not pass the genetic bottleneck yet; thus, they may contain multiple mtDNA variants [50]. 

This protocol has been claimed to be successfully implemented by two different groups, both performed in 2015, in a reference population of patients with a history of poor oocyte and embryo quality [43,44]. These groups claimed the efficiency of the AUGMENT^®^ technique by increasing fertilization and embryo quality [44], as well as pregnancy rates in comparison with a previous IVF treatment in the same patient [43].

Despite this, a well-designed pilot study performed in Spain in 2019 demonstrated the inability of the AUGMENT^®^ technique to improve the embryo development potential and pregnancy rates. This was a triple-blind, randomized, single-centre controlled experimental pilot study involving 57 poor-prognosis patients with previous IVF failures and well-documented poor embryo quality. In the same ovarian stimulation cycle for each patient, retrieved oocytes were randomized (1:1 ratio) to undergo standard ICSI or the AUGMENT^®^ protocol, which allows an intrapatient and intracycle comparison design to avoid any potential bias [42].

Despite the evident strength of the study design, several comments have been made since its publication. One of the main criticisms is that mitochondria are injected into meiosis II oocytes, while the majority of aneuploidies, and particularly trisomies, usually occur during meiosis I [35]. Hence, the AUGMENT treatment may be performed too late, reducing its potential benefit to the developing oocyte. Additionally, the injection of isolated, purified mitochondria may not be as beneficial as their injection in conjunction with other factors, which may also enhance oocyte quality [51].

### 3.2. Immature Oocytes

One of the main weak points of the AUGMENT^®^ technique is the use of cell types that have not yet passed the genetic bottleneck [50], significantly increasing the number of mtDNA variants. 

However, mitochondria isolated from immature oocytes have already passed this genetic bottleneck, and they can be obtained by several approaches: follicular in vitro activation, oocytes from cryopreserved tissue, residual immature oocytes from stimulated IVF cycles and immature oocytes from small follicles of less than 12–14 mm (which may not be usually punctured) [1]. 

Despite these promising sources of healthy mitochondria, no studies applying this technique in humans have yet been published. 

### 3.3. Granulosa Cells

Even though they do not share the same cell lineage, granulosa cells are the closest related cell type to the oocyte after ovarian stem cells and immature oocytes. However, their main advantage is the ease of their collection, as these cells are obtained at the same time as the oocyte during the follicular aspiration procedure. 

In 2004, this protocol significantly increased embryo quality (59.4% vs. 34.9% in the control group; *p* < 0.05) in a group of patients with a previous failed IVF treatment or older than 37 years [41], as well as pregnancy rates in patients with a previous failed IVF treatment [45]. 

### 3.4. Non-Ovarian Stem Cells

Mitochondria from granulosa cells undergo the ageing process along with the oocyte [11]. Hence, stem cells may be the ideal cell-type source of these organelles. In addition, mitochondria from stem cells resemble those from mature oocytes due to their similar metabolic adaptations [52], both being types of spherical mitochondria with few cristae [53]. 

Given the controversy about the existence of ovarian stem cells, stem cells from other lineages have been proposed as potential sources of autologous mitochondria.

One of the non-ovarian stem cells proposed is adipose-derived stem cells. Wang et al. have proven that adipose-derived stem cells mitochondria transfer rescues oocyte quality in aged mice [11]. Additionally, they did not see any morphological difference in mitochondria from these cells between young and aged mice, while they did see significant differences in mitochondria between young and aged oocytes [11]. On the contrary, Sheng et al. did not find any advantage of this technique in aged mice [54]. 

Unfortunately, there are no human studies using this technique in infertile patients. 

## 4. Discussion

Poor oocyte quality is a common cause of infertility in patients of advanced maternal age or with a history of previous IVF failure attempts. Problems at the cytoplasm level, and particularly in mitochondrial function, are among the main triggers of this condition [55,56]. Hence, mitochondria enrichment or replacement techniques may constitute effective therapeutic approaches to increase the chances of success in this type of patient. 

In this commentary, we have given an overview of the current state of mitochondria transfer techniques in the human oocyte. There are several options available, although at the moment, none of them has demonstrated great superiority over the others. Table 3 provide useful information regarding which techniques have been clinically used and which ones have yielded promising results in infertile patients. 

On the one hand, heterologous nuclear transfer techniques include a broad range of highly technologically demanding approaches, still under research, in which the main limitation is the generation of mtDNA heteroplasmy in the transferred oocyte [2,3]. Fortunately, the amount of mtDNA carryover has been significantly reduced over the years, and these technologies have been shown to overcome infertility in several couples with poor prognoses [24,27]. In contrast, heterologous ooplasmic transfer is a much easier technique to perform but always results in heteroplasmy [16,31,32].

Nevertheless, why mitochondria from a donor (which should be detected as foreign material) are not marked for destruction, as it happens with the sperm mitochondria during fertilization, remains unknown. Further molecular studies using heterologous mitochondria transfer should be performed following the traceability of the foreign mitochondria transferred in order to better understand how these mitochondria act and potentially enhance the patient’s oocyte quality. 

On the other hand, autologous transfer techniques always avoid heteroplasmy, as mitochondria are of autologous origin. In addition, mitochondria are transferred into the oocyte along with the spermatozoa during the ICSI procedure [41,42], thus simplifying the process. However, the technologically demanding step, in this case, is mitochondria isolation from a specific autologous cell type. 

In this context, stem cells are the most suitable cell-type source of autologous mitochondria. Stem cells share metabolic adaptations with the mature oocyte, thus having very similar mitochondria [53], without being subject to age. Ovarian stem cells have not been shown to improve reproductive outcomes in poor prognosis patients [42], while non-ovarian stem cells have not been tested in human studies. Likewise, mitochondria isolated from immature oocytes appear to be a promising option but have not been tested in human studies. Finally, mitochondria isolated from granulosa cells have been shown to improve embryo quality [41] and pregnancy rates [45], even though this cell type undergoes age along with the oocyte [11]. 

Therefore, further human studies using mitochondria transfer techniques are needed, especially of autologous origin. The optimization of these promising technologies may be a feasible option to increase the chances of success in infertile patients with poor oocyte quality. 

## 5. Conclusions

Currently, there is no mitochondria transfer technique with clear superiority over the rest. Autologous techniques may be the ideal approach, leaving no option for heteroplasmy. However, they have been poorly investigated in human studies. In contrast, heterologous approaches have been studied more extensively in humans, and the optimization of nuclear transfer techniques has succeeded in minimizing the amount of mtDNA carryover. In any case, the latter are highly technologically demanding approaches that need experienced hands in order to be able to obtain high success rates without damaging the oocyte. Nevertheless, given the high demand for new approaches to improve human oocyte quality, further clinical studies are needed in order to bring these techniques into regular practice and to give these patients a chance to achieve a pregnancy with their own oocytes. 

## Figures and Tables

**Table 1:** Main human clinical studies using heterologous mitochondria transfer.

Study Name	Type of Mitochondria Transfer	*n*	Patients’ Etiology	Main Outcome
Cohen, 1997 [15]	Ooplasmic transfer	1 patient	History of impaired embryo development	First human birth using this approach
Cohen, 1998 [13]		8 cycles	Repeated implantation failure	Improved results using the injection technique vs. electrofusion. One healthy infant and ongoing pregnancy in the injection group (total n = 5) vs. no pregnancy in the electrofusion group (n = 3)
Brenner, 2000 [16]		23 cycles		Twelve clinical pregnancies and overall improved embryo development. Proven mtDNA heteroplasmy in the offspring.
Huang, 1999 [17]		9 cycles		Five healthy infants after ooplasmic transfer from tripronucleated zygotes
Dale, 2001 [18]		1 patient		Birth of healthy twins
Chen, 2016 [19]		33 cycles		Follow-up study of 17 healthy infants from 13 couples [13,15,16]. Limited study with high bias, but overall unaffected healthy offspring.
Sobek, 2021 [20]		125 cycles Ooplasmic transfer vs. control in sibling oocytes	Low ovarian function	Increased fertilization and embryo development rates. A reduction in fertilization rates with age was observed in the control group but not in the ooplasmic transfer group. 28 healthy infants in the ooplasmic transfer group.
Zhang, 1999 [21]	GV transfer	60 GVs	Advanced maternal age	12 GVs were successfully removed, transferred, and fused into previously enucleated oocytes from young patients. 7 of these matured to a metaphase II oocyte, similar maturation rate to the non-manipulated GVs.
Darbandi, 2020 [22]		10 GVs		0% fusion rate
Tanaka, 2009 [23]	Spindle transfer	31 MII spindle transfer group 98 MII control group	In vitro matured MII oocytes (model of aged oocytes)	25/31 correctly fused (80.6%). Significantly higher number of oocytes developed to the blastocyst stage in the spindle transfer group (7 vs. 3 in the control group).
Zhang, 2017 [24]		1 patient	History of pregnancy loss and asymptomatic carrier of a Leigh syndrome mutation	First human birth after spindle transfer
Costa-Borges, 2020 [25]		9 cycles	Age range 32–40 years. History of embryo developmental arrest	Preliminary results from a larger pilot study (n = 25). Applied successfully in 39/44 oocytes (88.6%). Of these, 76.9% (30/39) fertilized and 20 developed into good quality blastocysts (66.7%). Genetic analysis revealed 35% (7/20) of the embryos to be euploid and mtDNA carryover levels <1%. Two blastocysts were warmed and transferred, resulting in two pregnancies.
Craven, 2010 [3]	PN transfer	80 uni- and tripronucleated zygotes with PN transfer vs. 76 unmanipulated control group	Transfer of PN from abnormally fertilized zygotes discarded from IVF cycles	First PN transfer attempt in humans. Minimal mtDNA carryover and compatible with onward development to the blastocyst stage.
Hyslop, 2016 [26]		523 MII	MII donated oocytes fertilized with donated sperm	Alternative approach based on transplanting pronuclei shortly after completion of meiosis rather than shortly before the first mitotic division. mtDNA carryover below 2%. Efficient development to the blastocyst stage with no detectable effect on aneuploidy or gene expression.
Zhang, 2016 [27]		1 patient	History of embryo developmental arrest	Viable pregnancy with normal karyotype and minimal mtDNA heteroplasmy
Ma, 2017 [28]	PB1 transfer	32 oocytes in PB1T group vs. 21 in the control group 11 women	Healthy volunteers	Oocytes supported the formation of de novo meiotic spindles and, after fertilization with sperm, meiosis completion and formation of normal diploid zygotes. Lower blastocyst formation rates in the PB1T group in comparison to the control group
Zhang, 2017 [29]	PB1 and PB2 transfer	1 patient	Repeated embryo fragmentation of maternal origin	PB1T but not PB2T into enucleated in vitro matured donor MII oocytes successfully generate normal fertilized zygotes with high efficiency for developing into blastocysts
Tang, 2019 [30]	PB2 transfer	134 oocytes	In vitro matured oocytes and in vivo matured oocytes with smooth endoplasmic reticulum aggregate, both donated from young women	Novel strategy for PB2 transfer. Unaltered blastocyst quality in the PB2T and control groups and similar euploidy rates

MII: metaphase II. PN: pronuclear. GV: germinal vesicle. PB: polar body. PBT: polar body transfer. mtDNA: mitochondrial DNA. *p* < 0.05 are considered statistically significant.

**Table 2:** Main human clinical studies using autologous mitochondria transfer.

Study Name	Type of Mitochondria Transfer	*n*	Patients’ Etiology	Main Outcome
Fakih, 2015 [43]	Ovarian stem cells (AUGMENT^®^)	59 + 34 patients (2 different clinics)	Poor oocyte and embryo quality	Poor study design with high bias. Increased pregnancy rates in comparison to the historic IVF success rates in the same patients
Oktay, 2015 [44]		16 patients	2 or more previous IVF attempts failure, and poor oocyte and embryo quality	Poor study design with high bias. Higher fertilization rates (78.3% vs. 47.9%; *p* = 0.036) and better embryo quality (3.1% vs. 2.3%; *p* = 0.082) than the results obtained in previous cycles from the same patients.
Labarta, 2019 [42]		57 patients	Previous IVF failures and well-documented poor embryo quality	Intrapatient and intracycle comparison design. Significantly lower day 5 blastocyst formation rate in the AUGMENT group. No statistically significant differences in any other variable studied.
Kong, 2004 [41]	Granulosa cells	18 patients	A previous failed IVF treatment or order than 37 years	Similar fertilization rates (74.4% vs. 76.8% in the control group; *p* > 0.05). Significantly higher good quality embryo rate in mitochondria transfer group (59.4% vs. 34.9% in the control group; *p* < 0.05). There were 7 clinical pregnancies in the 18 cases.
Tzeng, 2004 [45]		71 cycles vs. 81 historic cycles in the same patient group	A previous failed IVF treatment	Significantly higher pregnancy rates (35.2% vs. 6.2% in the historic control group; *p* < 0.05) and lower miscarriage rates (15.4% vs. 100% in the historic control group; *p* < 0.05). Significantly higher day 3 embryo quality. Twenty live births. Oocytes following this technique had a propensity to cleave faster, as well as lower apoptosis and fragmentation rates.

*p* < 0.05 are considered statistically significant.

**Table 3:** Summary table of the clinical use in infertile patients and the main results of the different techniques here described. Clinical use is defined as:

Type of Mitochondria Transfer	Clinically Used in Infertile Patients (Yes/No)	Has Showed Promising Results (Yes/No)	Live Birth/s (Yes/No)
Ooplasmic transfer	Yes	Yes	Yes
Germinal vesicle transfer	Yes	Yes	No
Spindle transfer	Yes	Yes	Yes
Pronuclear transfer	Yes	Yes	Yes
First polar body transfer	Yes	Yes	No
Second polar body transfer	Yes	No	No
Ovarian stem cells	Yes	No	Yes
Immature oocytes	No	-	-
Granulosa cells	Yes	Yes	Yes
Non-ovarian stem cells	No	-	-
